# Psychosocial factors mediate social inequalities in health-related quality of life among children and adolescents

**DOI:** 10.1186/s12889-024-20393-0

**Published:** 2024-10-29

**Authors:** Viviane Richard, Elsa Lorthe, Roxane Dumont, Andrea Loizeau, Hélène Baysson, Stephanie Schrempft, María-Eugenia Zaballa, Julien Lamour, Rémy P. Barbe, Klara M. Posfay-Barbe, Idris Guessous, Silvia Stringhini

**Affiliations:** 1 Unit of Population Epidemiology, Division of Primary Care Medicine, Geneva University Hospitals, Rue Jean-Violette 29, Geneva, 1205 Switzerland; 2 Department of Health and Community Medicine, Faculty of Medicine, University of Geneva, Michel Servet 1, Geneva, 1211 Switzerland; 3 Centre for Research in Epidemiology and Statistics Paris (CRESS), Université Paris Cité, Inserm, INRAE, 147 Rue de l’Université, Paris, 75007 France; 4 Division of Child and Adolescent Psychiatry, Department of Woman, Child, and Adolescent Medicine, Geneva University Hospitals, Rue Gabrielle-Perret-Gentil 4, Geneva, 1205 Switzerland; 5 Pediatric Infectious Disease Unit, Department of Pediatrics, Gynecology & Obstetrics, Geneva University Hospitals and Faculty of Medicine, Rue Gabrielle-Perret-Gentil 4, Geneva, 1205 Switzerland; 6 Division of Primary Care Medicine, Geneva University Hospitals, Rue Gabrielle-Perret-Gentil 4, Geneva, 1205 Switzerland; 7 School of Population and Public Health and Edwin S.H, Leong Centre for Healthy Aging, Faculty of Medicine, University of British Columbia, 117-2194 Health Sciences Mall, Vancouver, BC V6T 1Z3 Canada

**Keywords:** Health behaviours, Psychosocial, Health-related quality of life, Socio-economic inequalities, Mediation

## Abstract

**Background:**

The present analysis aimed to assess the mediating role of psychosocial and behavioural factors in socio-economic inequalities in health-related quality of life (HRQoL) among children and adolescents.

**Methods:**

Cross-sectional data was drawn from the randomly selected SEROCoV-KIDS cohort study in Geneva, Switzerland. Associations of socio-economic conditions (parents’ highest education, household financial situation) with HRQoL, psychosocial (parent–child relationship, school difficulties, friends, extracurricular activities) and behavioural factors (screen time, physical activity, green spaces time, sleep duration), along with associations of psychosocial and behavioural factors with HRQoL, were evaluated with generalized estimating equations. Counterfactual mediation analyses were conducted to test pathways linking socio-economic conditions to HRQoL.

**Results:**

Of 965 children and 816 adolescents, those with disadvantaged financial circumstances were more likely to have a poor HRQoL (adjusted Odds Ratio [aOR]: 3.80; 95% confidence interval [CI]: 1.96–7.36 and aOR: 3.66; 95%CI: 2.06–6.52, respectively). Psychosocial characteristics mediated 25% (95%CI: 5–70%) and 40% (95%CI: 18–63%) of financial disparities in HRQoL among children and adolescents, respectively. Health behaviours were weakly patterned by socio-economic conditions and did not contribute to financial differences in HRQoL.

**Conclusions:**

These findings provide empirical evidence for mechanisms explaining socio-economic disparities in child HRQoL and could inform interventions aimed to tackle health inequalities.

**Supplementary Information:**

The online version contains supplementary material available at 10.1186/s12889-024-20393-0.

## Background

Disadvantaged socio-economic conditions are associated with poorer health outcomes among children and adolescents [1]. This relationship takes the form of a gradient and has consistently been found for different physical and mental health conditions [1]. Examples range from asthma to attention deficit hyperactivity disorder [2, 3], and also include broader subjective health outcomes such as health-related quality of life (HRQoL) [4, 5], a widely used indicator of overall well-being reflecting individual’s perception of physical, emotional and social health [6].

Psychosocial and behavioural pathways have commonly been proposed to explain socio-economic inequalities in health [7]. Children and adolescents from disadvantaged backgrounds are at increased risk of feeling a lack of control over their life and of having limited interpersonal resources to rely on [7]. Furthermore, research has shown that they experience reduced social participation [8], increased school difficulties [9] and family strain [10]. Moreover, despite some inconsistent evidence [11, 12], systematic reviews showed that children and adolescents with adverse socio-economic conditions tend to engage more often in unhealthy behaviours, including excessive recreational screen time [13], low level of physical activity [14] and short sleep duration [15]. In turn, psychosocial and behavioural risk factors are generally related to worse HRQoL [16, 17].

There is ample evidence for a meaningful contribution of psychosocial and behavioural factors in socio-economic disparities in health and HRQoL among adults [18–20] and adolescents [16, 21–23]. Among children, previous reports show that socio-economic inequalities in HRQoL could partially be explained by variations in parenting practices and parental mental health [24, 25]. Finally, one study conducted in China found that dietary behaviours and the home literacy environment mediated the association between socio-economic conditions and quality of life among participants aged 9 to 14 years old [26]. However, to the best of our knowledge, research examining both psychosocial and behavioural mediators of socio-economic disparities in HRQoL specifically among children is lacking. Yet, focusing on children and adolescents separately is important since explanatory patterns could differ [27]. A detailed understanding of the mechanisms by which socio-economic circumstances shape children’s and adolescents’ HRQoL is needed as it could support public health decision-makers and inform the design of relevant interventions. Beyond young people’s current well-being, this is of particular relevance given that childhood experiences influence later health outcomes [7].

In this context, we aimed to examine the extent to which psychosocial factors and health behaviours mediate the association between socio-economic conditions and HRQoL. We used a population-based sample of children and adolescents from Geneva, Switzerland, which is characterized by good public education, low unemployment rate, comparatively high quality of life, and yet persistent socio-economic inequalities in health [5, 28]. Given evidence among adults and adolescents, we hypothesised that psychosocial and behavioural factors would partially mediate this association.

## Methods

### Study design

Data was drawn from the baseline assessment of the SEROCoV-KIDS cohort study and from a COVID-19 serosurvey [29], which shared similar recruitment and data collection procedures. In both studies, inclusion criteria were 1) to be aged between 6 months and 17 years old, 2) to live in the canton of Geneva, Switzerland, and 3) to have been (or have a sibling) randomly selected from population registries, or to be part of the household of a randomly selected individual for previous studies conducted by our research group. Incentive for participation included a free anti-SARS-CoV-2 serological testing. The Geneva Cantonal Commission for Research Ethics approved the study. One referent parent (or legal guardian) per household, as well as adolescents aged 14 years or older provided written informed consent to participate; children gave oral assent.

Data collection took place between December 2021 and June 2022. Referent parents completed a health and lifestyle questionnaire on behalf of each of their participating children, as well as a health and socio-demographic questionnaire about themselves and their household. Participants aged 14 years and older were additionally invited to complete a health and a lifestyle questionnaire specifically tailored for their age group. Data was collected through the online and secure platform specchio-hub.ch.

### Analytical sample

We restricted the current analyses to children and adolescents aged 6 to 17 years because some psychosocial data under study (e.g. school difficulties, number of friends) were not collected in younger SEROCoV-KIDS participants. Additionally, four individuals whose sex was reported as *other* were excluded because of the low number of occurrence and one was excluded due to missing data on all health behaviours. The final sample included 1781 participants and was stratified into children aged 6–11 years old (n = 965) and adolescents aged 12–17 years old (n = 816) to assess differences according to age (Supplementary Fig. 1).

### Conceptual approach

**Fig. 1:**
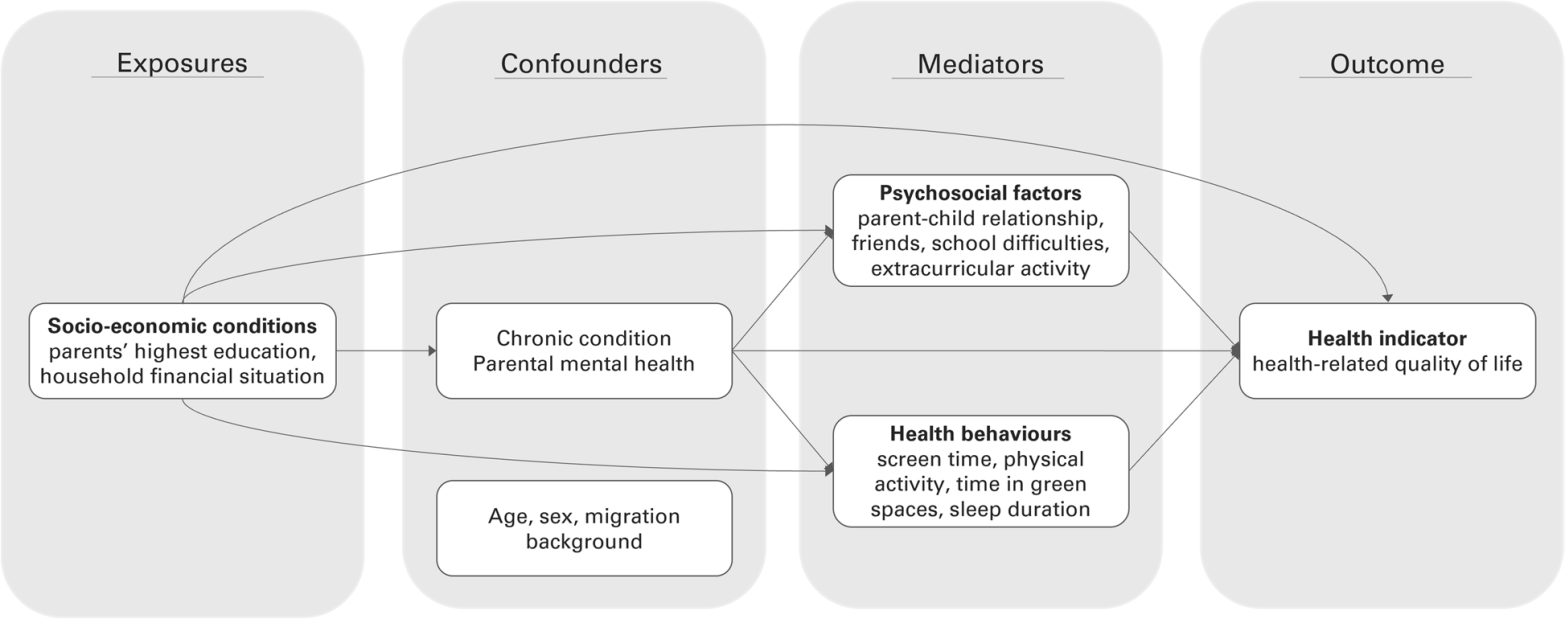
Directed acyclic graph (DAG) of the association between socio-economic conditions, psychosocial factors, health behaviours and health indicator. Dashed lines indicate confounding pathways. Age, sex and migration background are considered to be covariates of all associations; arrows are not drawn for readability. DAG of the models with psychosocial and behavioural mediators only are presented in Supplementary Figs. 2 and 3, respectively

To guide variable choice and modelling decisions, hypothesised pathways between socio-economic exposures, psychosocial and behavioural mediators, HRQoL outcome, and their confounders were drawn in a directed acyclic graph (DAG) based on a scoping literature review (Fig. 1). Socio-economic conditions were described with parental education and household financial situation to capture both social and economic aspects of disadvantage [7]. HRQoL was chosen as a widely recognized multidimensional construct reflecting individual’s perception of overall well-being [6]. Psychosocial and behavioural mediators were selected based on previous report to encompass a broad range of children’s functioning and habits across different areas of life [16]. Confounders were of two types: socio-demographic factors (age, sex, migration background), considered to be covariates of all associations, and confounders of the mediator—outcome relationship (chronic condition, parental mental health), assumed to be affected by the socio-economic exposure according to the social causation hypothesis [30].

### Measures

The conceptual approach was operationalized with the following variables reported by the referent parent, unless otherwise stated.

#### Exposures – socio-economic conditions

The parents’ highest education was obtained from combining the educational level (“college” vs "lower than college”) of both parents. In case of single parent household or of missing data on the second parent, only the education of the referent parent was considered. The household financial situation was categorized into “very good”, if parents had a comfortable financial situation in which they could easily save money, and “good” if they could cover their needs and face minor unexpected expenses. Due to the low number of occurrences, answers such as “I have to be careful with my expenses, and unexpected expenses could put me into financial difficulties” or “I am not able to cover my needs with my income and I need external support” were grouped into an “average-to-poor” category.

#### Mediators – psychosocial factors and health behaviours

We considered the following psychosocial factors, covering family, social and educational dimensions, namely parent–child relationship (“good” vs “less than good”), the number of close friends (“two or more” vs “one or none”), school difficulties (“no” vs “yes”), and engagement in any extracurricular activity (“yes” vs “no”). Because of the small number of observations in some categories, variables were dichotomised as described in the Supplementary Table 1.

Regarding health behaviours, leisure screen time, physical activity and sleep duration were reported in hours per day by parents (self-report of screen time from 14 years old) for both week and weekend days. For each behaviour, the average time per day was calculated as: (week time $\times$ 5 + weekend time $\times$ 2) / 7. Adherence to behaviour recommendations was then defined using age-specific thresholds from the World Health Organization’s guidelines [31], when available, or from the Canadian 24-Hour Movement Guidelines otherwise [32], as detailed in the Supplementary Table 1. Time spent in green spaces was collected in hours per week and dichotomized by combining the middle and highest tertiles to form an “average to high” category in comparison to the lowest tertile, as no guideline was found (Supplementary Table 1).

#### Outcome – health-related quality of life

HRQoL was measured with the French version of the Pediatric Quality of Life Inventory (PedsQL) Short Form [33]. The scale was parent-reported for children aged 6 to 13 years, and self-reported for those aged 14 years and older. The total score had a good internal consistency (α = 0.85) and was dichotomized using published thresholds to identify individuals with a poor HRQoL and provide clinically interpretable estimates [33] (Supplementary Table 1).

#### Confounders

Age, sex, migration background, chronic condition, and parental mental health were additionally included. Migration background was characterized by the parents’ country of birth and dichotomized into “At least one parent born in Switzerland” vs “Parents born abroad”. In case of single parent household or of missing data, only the country of birth of the referent parent was considered. Child chronic condition was defined by the reported diagnosis of a physical or mental behavioural and neurodevelopmental disorder lasting more than six months. The self-reported mental health of the referent parent was dichotomized into “good” (“very good” or “good”) vs “average-to-poor” (“average”, “poor” or “very poor”).

### Statistical analyses

The exposures – outcome, exposure – mediators and mediators – outcome associations were evaluated with generalized estimating equations (GEE). This approach enabled to account for the clustering of data within households as some participants were siblings (number of households: 735 and 629, mean number of included children per household [standard deviation]: 1.31 [0.51] and 1.30 [0.50] in the children and adolescents samples, respectively). GEE were defined as following a binomial distribution and parametrized with an exchangeable covariance matrix using the R geepack package [34]. Following the DAG (Fig. 1), the associations of socio-economic conditions with psychosocial factors and health behaviours, as well as with HRQoL were controlled for age, sex and migration background, while the regression of HRQoL on psychosocial and behavioural factors was further adjusted for chronic condition and parental mental health. Assuming variables missing at random, multiple imputation was performed by chained equations with 10 repetitions using the R mice package [35], and with all the above-mentioned variables as predictors.

Counterfactual mediation analyses of the relationship between socio-economic conditions and HRQoL were conducted for each exposure (parents’ highest education, household financial situation) and with each group of mediators (psychosocial, behavioural, all), without assuming any specific pathways between the mediators. The use of counterfactual approach for mediation analysis has been recommended in recent literature as it relies on general definitions of direct and indirect effects that hold in case of non-linearity or interactions [36]. Logistic marginal structural models proposed by VanderWeele and Tchetgen Tchetgen [37] and implemented in the the R CMAverse package [38] were used to estimate direct, indirect and total effects, as well as the proportion mediated calculated as: direct effect $\times$ (indirect effect – 1) / (total effect – 1). This approach was used because it enables to relax the often unrealistic assumption of absence of mediator—outcome confounders affected by the exposure, by explicitly including them [37]. However, this method can only handle categorical mediators, hence the dichotomization of continuous behavioural mediators. Missing data was handled with multiple imputation using the built-in function of the package and confidence intervals calculated using bootstraps with 1000 repetitions. In sensitivity analyses, E-values were calculated to estimate the minimal strength of association that an unmeasured confounder should have with both the exposure and outcome to explain away the observed effect.

All analyses were stratified by age to account for the fact that the associations under study may vary depending on the age group [27]. Estimations were performed with R 4.2.2.

## Results

Analyses included 965 children aged 6–11 years old (mean age: 8.7 years, 47.6% girls) and 816 adolescents aged 12–17 years old (mean age: 14.3 years; 52.1% girls). Twelve percent of individuals had at least one missing information and variable missingness ranged from 0 to 8% (Table 1). Overall, 9.4% of children and 16.7% of adolescents had a poor HRQoL. The distribution of psychosocial and behavioural factors also varied according to age group, adolescents generally displaying less favourable characteristics (Table 1).

**Table 1:** Socioeconomic, psychosocial, behavioural, and health characteristics of children and adolescents

	**Children** **6–11 years old** **(*n* = 965)**	**Adolescents** **12–17 years old** **(*n* = 816)**
	n (%)	n (%)
Socioenomic conditions		
**Parents’ highest education**		
College or higher	805 (83.7)	652 (80.0)
Lower than college	157 (16.3)	163 (20.0)
Missing	3	1
**Household financial situation**		
Very good	326 (36.8)	275 (36.4)
Good	391 (44.1)	335 (44.4)
Average to poor	170 (19.2)	145 (19.2)
Missing^a^	78	61
Psychosocial factors		
**Parent–child relationship**		
Good	811 (84.0)	623 (76.3)
Less than good	154 (16.0)	193 (23.7)
**Friends**		
Two or more	757 (78.7)	641 (79.0)
One or none	205 (21.3)	170 (21.0)
Missing	3	5
**School difficulties**		
No	907 (94.2)	726 (89.0)
Yes	56 (5.8)	90 (11.0)
Missing	2	0
**Extracurricular activity**		
Yes	915 (94.8)	683 (83.7)
No	50 (5.2)	133 (16.3)
Health behaviours		
**Leisure screen time**		
Meets recommendations	867 (89.9)	310 (40.3)
Does not meet recommendations	97 (10.1)	460 (59.7)
Missing	1	46
**Physical activity**		
Meets recommendations	706 (73.3)	469 (57.6)
Does not meet recommendations	257 (26.7)	345 (42.4)
Missing	2	2
**Green spaces time**		
Average to long	633 (66.4)	333 (41.3)
Little	320 (33.6)	474 (58.7)
Missing	12	9
**Sleep duration**		
Meets recommendations	819 (85.0)	571 (70.0)
Does not meet recommendations	144 (15.0)	245 (30.0)
Missing	2	0
Health outcome		
**Health-related quality of life**		
Good	873 (90.6)	642 (83.3)
Poor	91 (9.4)	129 (16.7)
Missing	1	45

^a^ Among which, 69 and 58, respectively, chose the option “I do not wish to answer”

Compared with a very good household financial situation, average-to-poor circumstances were associated with a poor HRQoL among both children (adjusted odds ratio [aOR]: 3.80; 95% Confidence Interval [CI]: 1.96–7.36) and adolescents (aOR: 3.66; 95% CI: 2.06–6.52), while good circumstances were only related to a poor HRQoL among children (aOR: 2.41; 95% CI: 1.29–4.49, Table 2). A lower parental education was a risk factor for a poor HRQoL among children (aOR: 2.96; 95% CI: 1.80–4.89), but not among adolescents (aOR: 1.54; 95% CI: 0.95–2.50).

**Table 2:** Association of socio-economic conditions with health-related quality of life, psychosocial risk factors and health behaviours

	**Health indicator**	**Psychosocial risk factors**				**Health behaviours**				
	Poor health-related quality of life	Less than good parent–child relationship	One or no friends	Presence of school difficulties	No extracurricular activity	Not meeting screen time recommendation	Not meeting physical activity recommendation		Little green spaces time	Not meeting sleep recommendation
	aOR (95% CI)	aOR (95% CI)	aOR (95% CI)	aOR (95% CI)	aOR (95% CI)	aOR (95% CI)	aOR (95% CI)		aOR (95% CI)	aOR (95% CI)
Children 6-11 years old (*n* = 965)										
**Parents’ highest education**										
College	Ref	Ref	Ref	Ref	Ref	Ref	Ref		Ref	Ref
Lower than college	2.96 (1.80—4.89)	1.15 (0.71—1.84)	1.72 (1.14—2.61)	2.63 (1.45—4.78)	4.28 (2.24—8.21)	2.63 (1.55—4.45)	0.83 (0.54—1.26)		1.03 (0.70—1.53)	1.70 (1.08—2.68)
**Household financial situation**										
Very good	Ref	Ref	Ref	Ref	Ref	Ref	Ref		Ref	Ref
Good	2.41 (1.29—4.49)	1.07 (0.71—1.63)	1.22 (0.81—1.85)	0.71 (0.33—1.51)	4.75 (1.38—16.40)	1.28 (0.75—2.18)	0.99 (0.69—1.43)		1.07 (0.73—1.54)	0.66 (0.40—1.09)
Average-to-poor	3.80 (1.96—7.36)	1.02 (0.60—1.72)	2.21 (1.39—3.50)	2.01 (1.00—4.04)	8.01 (2.16—29.68)	1.70 (0.93—3.10)	0.99 (0.64—1.53)		1.08 (0.69—1.70)	1.78 (1.10—2.89)
Adolescents 12-17 years old (*n* = 816)										
**Parents' highest education**										
College	Ref	Ref	Ref	Ref	Ref	Ref	Ref		Ref	Ref
Lower than college	1.54 (0.95—2.50)	1.52 (1.00—2.30)	1.53 (1.01—2.31)	1.94 (1.17—3.24)	4.57 (2.93—7.14)	1.39 (0.94—2.05)	1.38 (0.96—1.99)		1.21 (0.83—1.79)	1.20 (0.81—1.77)
**Household financial situation**										
Very good	Ref	Ref	Ref	Ref	Ref	Ref	Ref	Ref		Ref
Good	1.56 (0.96—2.52)	1.40 (0.92—2.12)	1.45 (0.95—2.21)	2.07 (1.13—3.79)	1.95 (1.17—3.25)	1.37 (0.95—1.96)	1.56 (1.10—2.24)	1.35 (0.94—1.95)		0.97 (0.67—1.42)
Average-to-poor	3.66 (2.06—6.52)	1.48 (0.89—2.45)	2.04 (1.22—3.40)	4.09 (2.10—7.96)	3.88 (2.17—6.94)	1.10 (0.67—1.81)	1.51 (0.97—2.35)	1.44 (0.91—2.26)		1.08 (0.69—1.71)

Results are adjusted odds ratios (aOR) and 95% confidence intervals (CI) from generalized estimating equations following a binomial distribution adjusted for age, sex and migration background. Missing data are imputed by chained equations

Children living in households with an average-to-poor financial situation were more likely to display psychosocial risk factors, such as few close friends (aOR: 3.80; 95% CI: 1.96–7.36), school difficulties (aOR: 2.01; 95% CI: 1.00–4.04), and no engagement in extracurricular activities (aOR: 8.01; 95% CI: 2.16–29.68, Table 2). Similar trends were observed among adolescents (Table 2). Results concerning health behaviours were mixed; an average-to-poor financial situation was solely related to a lower adherence to sleep guidelines among children (aOR: 1.78; 95% CI: 1.10—2.89). Patterns were similar when examining the parents’ highest education as socio-economic indicator (Table 2).

Finally, except for school difficulties among children, all psychosocial risk factors were associated with a poor HRQoL in both age groups (Table 3). Children not meeting sleep duration guidelines (aOR: 1.84; 95% CI: 1.08–3.14), as well as adolescents not adhering to screen time (aOR: 2.33; 95% CI: 1.39–3.91) and physical activity recommendations (aOR: 1.52; 95% CI: 1.00–2.31) were also more likely to experience a poor HRQoL (Table 3).

**Table 3:** Association of psychosocial risk factors and health behaviours with health-related quality of life

	**Poor health-related quality of life**	
	**Children** **6–11 years old (*n* = 965)**	**Adolescents** **12–17 years old (*n* = 816)**
	aOR (95% CI)	aOR (95% CI)
Psychosocial risk factors		
**Parent–child relationship**		
Good	Ref	Ref
Less than good	2.02 (1.17—3.47)	2.91 (1.84—4.63)
**Number of friends**		
Two or more	Ref	Ref
One or none	1.78 (1.12—2.82)	1.74 (1.09—2.77)
**School difficulties**		
No	Ref	Ref
Yes	1.96 (0.93—4.13)	2.63 (1.53—4.52)
**Engagement in extracurricular activity**		
Yes	Ref	Ref
No	4.77 (2.43—9.37)	2.03 (1.20—3.44)
Health behaviours		
**Recreational screen time**		
Meets the recommendations	Ref	Ref
Does not meet the recommendations	1.44 (0.78—2.68)	2.33 (1.39—3.91)
**Physical activity**		
Meets the recommendations	Ref	Ref
Does not meet the recommendations	1.46 (0.90—2.39)	1.52 (1.00—2.31)
**Green spaces time**		
Average to high	Ref	Ref
Low	1.31 (0.81—2.12)	1.22 (0.78—1.88)
**Sleep duration**		
Meets the recommendations	Ref	Ref
Does not meet the recommendations	1.84 (1.08—3.14)	1.27 (0.83—1.96)

Results are adjusted Odds Ratios (aOR) and 95% confidence intervals (CI) from generalized estimating equations model following a binomial distribution, adjusted for age, sex, migration background, child’s lasting medical condition and parental mental health. Missing data are imputed by chained equations

Socio-economic conditions were associated with HRQoL as well as with psychosocial risk factors that in turn also influenced HRQoL, which provided the rationale for conducting mediation analyses. Support for a pathway linking socio-economic conditions and HRQoL through health behaviours was not consistent but we still decided to explore it further with formal mediation analyses.

**Fig. 2:**
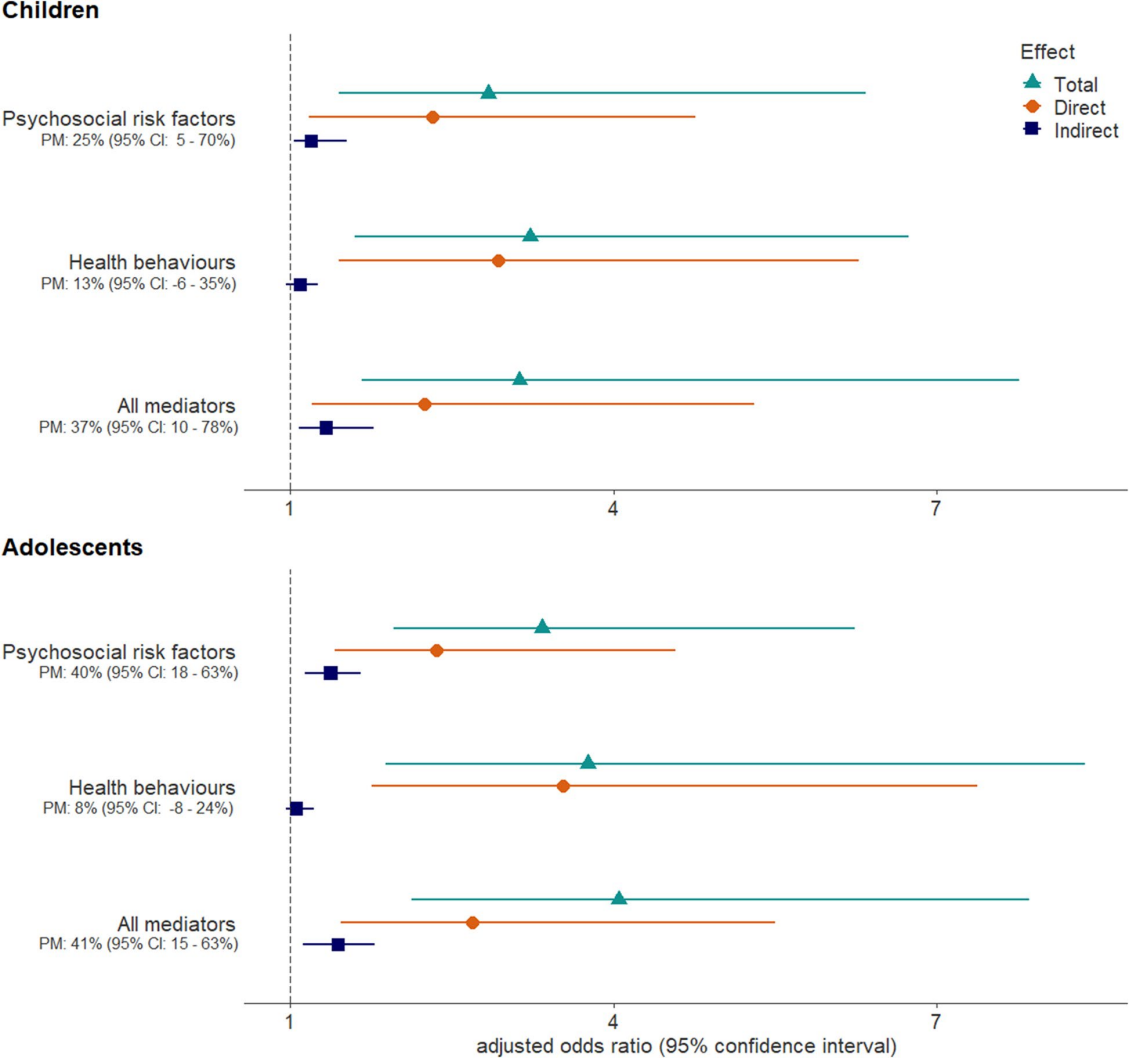
Mediation of psychosocial risk factors and health behaviours in the association between household financial situation and health-related quality of life among children (6–11 years old) and adolescents (12–17 years old). PM: proportion of the total effect mediated by the indirect effect of psychosocial and/or behavioural factors. Results are from logistic marginal structural models adjusted for age, sex and migration background, as well as for chronic condition and parental mental health as mediator—outcome confounders affected by the exposure

The counterfactual mediation analyses showed that the direct effect of socio-economic conditions on HRQoL was larger than their indirect effects through psychosocial or behavioural mediators (Fig. 2, Supplementary Table 2). Among children, psychosocial risk factors explained 25% (95% CI: 5–70%) of the association between the household financial situation and HRQoL; the combination of psychosocial and behavioural factors mediated 37% (95% CI: 10–78%) of the association (Fig. 2). Among adolescents, psychosocial risk factors accounted for 40% (95% CI: 18–63%) of the financial differences in HRQoL, while the indirect effect of health behaviours was not significant. When examining the parents’ highest education as socio-economic indicator, differences in HRQoL were only explained by psychosocial factors among children (proportion mediated: 26%; 95% CI: 8–45%, Supplementary Table 2). In sensitivity analyses, E-values of the total, direct and indirect effects ranged between 1.17 and 7.57 on the odds ratio scale. This corresponded to the minimal effect size that an unmeasured confounder should have for the observed effects to be explained away (Supplementary Table 2).

## Discussion

The present analyses showed that the greater the socio-economic disadvantage, the higher the risk of experiencing a poor health-related quality of life in children and adolescents. Part of the disparities found in our sample were explained by differences in psychosocial risk factors. Health behaviours did not seem to mediate socio-economic disparities in health-related quality of life.

The observed adverse effect of disadvantaged socio-economic conditions on the health of young people was in line with the existing body of literature [1]. It is worth noting that this study took place two years after the onset of the COVID-19 pandemic. Although sanitary measures were relatively light in Switzerland [39] and most of them were lifted at that time, it might reflect a situation with exacerbated health inequalities. Indeed, we previously showed that children with social and family vulnerabilities were particularly impacted by the pandemic and related restrictions, which in turn was associated with poorer health outcomes [40]. Interestingly, an average-to-poor financial situation was associated with a poor quality of life in both age groups, while this was only true for younger children when examining the parental education as socio-economic indicator. This might be related to the decreasing influence of parental education on adolescents as they pursue their own education, even though they continue to rely on their parents financially [41]. It supports the idea that the effect of a disadvantaged background on young people’s health may vary according to age [27].

We showed that psychosocial risk factors mediated about 25% of socio-economic inequalities in health-related quality of life among children, while health behaviours did not play a role. To the best of our knowledge, these are the first analyses of this kind focused specifically on children [16, 26]. Our findings thus contribute to a more comprehensive understanding of health inequalities by providing empirical evidence for the pathways linking socio-economic conditions to quality of life among children [7]. Additionally, we found that psychosocial characteristics explained 40% of financial disparities in health-related quality of life among adolescents, which was similar to the 39% found by Moor et al. [16] who focused on economic inequalities in self-rated health among adolescents with similar mediators. This relatively large contribution of psychosocial risk factors to subjective health disparities might be explained by the importance of social and school dimensions for young people’s life [42].

In our sample, health behaviours were not strongly patterned by socio-economic conditions, which echoes previous conclusions from systematic reviews [11, 12]. Furthermore, they were only weakly related to health-related quality of life. This reflects context-dependent findings from a cross-national study in which meeting screen time, physical activity and sleep duration recommendations was related to a better health-related quality of life in Australia and in Canada, but not in Finland and in Portugal. [17]. These weak and inconsistent associations explain why health behaviours did not mediate socio-economic differences in quality of life in our setting. Conversely, existing studies found a significant role of health behaviours in general health inequalities [18, 20, 21]. Apart from the different context, it could be because of the inclusion of additional important mediators. For example, Moor et al. [16] found the contribution of fruits and vegetables intake to be relatively large among adolescents, while we did not assess dietary habits. Smoking and alcohol consumption have also been identified as key behaviours to explain the socio-economic gradient in health among adults, but was less relevant in our paediatric setting [18]. Finally, it could be that socio-economic differences in health behaviours increase in adulthood [43], and that their effect on health accumulates over time, which could explain why findings are much clearer among adults [18].

Between 60 and 75% of socio-economic inequalities in health-related quality of life of children and adolescents remained unexplained by the putative mechanisms examined, which suggests the existence of other meaningful pathways. Beyond the unassessed health behaviours mentioned above, other non-included psychosocial characteristics could have played a role. For example, Straatmann et al. [44] observed that adverse childhood experiences prospectively mediated the association of socio-economic conditions at birth with behavioural problems, cognitive disability and excess weight at 14 years old. Cognitive characteristics, such as pessimism, were also shown to explain part of the relationship between socio-economic conditions and children’s emotional problems [45]. Other pathways including environmental exposures could also be considered. Hence, disadvantaged circumstances were shown to be a risk factor for poor housing and neighborhood quality, which in turn was associated with a poorer self-rated health [46].

Our findings should be interpreted in light of their limitations. First, our analyses relied mostly on parent-reported data, as it is generally the case in large scale population-based studies on children [47]. Highly educated individuals may be more prone to social desirability bias [48] and may overreport valued social and behavioural factors to a greater extent, leading to an overestimation of the mediating effects. Second, the temporality underlying the mediation could not be assessed due to the cross-sectional nature of data and associations may be reverse. For example, a good quality of life could give the opportunity for positive social interactions and healthy behaviours, or poor parental mental health could negatively impact the household socioeconomic circumstances. However, this is not expected to alter the effect of family socio-economic conditions on children, since they are likely to occur before the development of their psychosocial, behavioural and quality of life characteristics. Finally, the method used for the mediation analyses did not enable to estimate the effect of each individual mediator nor to account for the household clustering of participants. Yet, it is reassuring to note that the conclusions were coherent with our expectations based on the results of the GEE that included the household effect. The study also presents major strengths, including the random selection of a sizeable sample of young people encompassing a large age range, which enhances the generalizability of our results. The inclusion of multiple psychosocial and behavioural indicators as mediators, as well as the statistical adjustment for relevant mediator – outcome confounders affected by the exposure further adds to the robustness of our findings.

## Conclusion

Socio-economically disadvantaged children and adolescents had a poorer quality of life than their more privileged counterparts, which was partly explained by differences in psychosocial risk factors. These findings provide empirical evidence supporting theorized pathways to socio-economic disparities in child health, and give insight into promising areas of interventions to tackle children’s inequalities in quality of life.

Although structural changes are paramount to tackle the roots of health inequalities [49], this study indicates that psychosocial aspects could represent an effective target for public policies and interventions addressing well-being equity among young people [50]. Measures may for example focus on providing children and adolescents with various opportunities for social engagement and enabling the development of positive school and family environments. However, there is no one-size-fits-all solution and interventions should be developed in partnership with the communities they serve to ensure they are contextually appropriate and relevant [51]. Finally, following the principle of proportionate universalism, such policies should aim at improving the quality of life over the whole socio-economic gradient by targeting all children but with varying intensity according to the needs to improve both their present and future health [49].

## Supplementary Information

Supplementary Material 1.

## Data Availability

The datasets used during the current study are available from the corresponding author on reasonable request.

## Abbreviations

aOR: Adjusted Odds Ratio
CI: Confidence interval
DAG: Directed acyclic graph
GEE: Generalised estimating equations
HRQoL: Health-related quality of life
PM: Proportion mediated
