# Supplementary material for: Psychosocial factors mediate social inequalities in health-related quality of life among children and adolescents
Source: BMC Public Health. 2024 Oct 29;24:2986. doi: 10.1186/s12889-024-20393-0 (PMC11520694; doi:10.1186/s12889-024-20393-0)
Supplement: Supplementary file 1 — Supplementary Material 1. [file 12889_2024_20393_MOESM1_ESM.pdf]

Psychosocial factors mediate social inequalities in health-related quality of life among children and adolescents

Supplementary material – BMC Public Health

Viviane Richard[a,b], Elsa Lorthe[a,c], Roxane Dumont[a,b], Andrea Loizeau[a], Hélène Baysson[a], Stephanie Schrempft[a], María-Eugenia Zaballa[a], Julien Lamour[a], Rémy P. Barbe[d], Klara M. Posfay-Barbe[e], Idris Guessous[b,f], Silvia Stringhini[a,b,g]; for the SEROCoV-KIDS study group

[a] Unit of Population Epidemiology, Division of Primary Care Medicine, Geneva University Hospitals, Jean-Violette 29, 1205 Genève, Switzerland.

[b] Department of Health and Community Medicine, Faculty of Medicine, University of Geneva, Michel Servet 1, 1211 Genève, Switzerland.

[c] Centre for Research in Epidemiology and Statistics Paris (CRESS), Université Paris Cité, Inserm, INRAE, 147 rue de l'Université, 75007 Paris, France.

[d] Division of Child and Adolescent Psychiatry, Department of Woman, Child, and Adolescent Medicine, Geneva University Hospitals, Rue Gabrielle-Perret-Gentil 4, 1205 Genève, Switzerland.

[e] Pediatric Infectious Disease Unit, Department of Pediatrics, Gynecology & Obstetrics, Geneva University Hospitals and Faculty of Medicine, Rue Gabrielle-Perret-Gentil 4, 1205 Genève, Switzerland.

[f] Division of Primary Care Medicine, Geneva University Hospitals, Rue Gabrielle-Perret-Gentil 4, 1205 Genève, Switzerland.

[g] School of Population and Public Health and Edwin S.H. Leong Centre for Healthy Aging, Faculty of Medicine, University of British Columbia, 117-2194 Health Sciences Mall, Vancouver, BC Canada V6T 1Z3

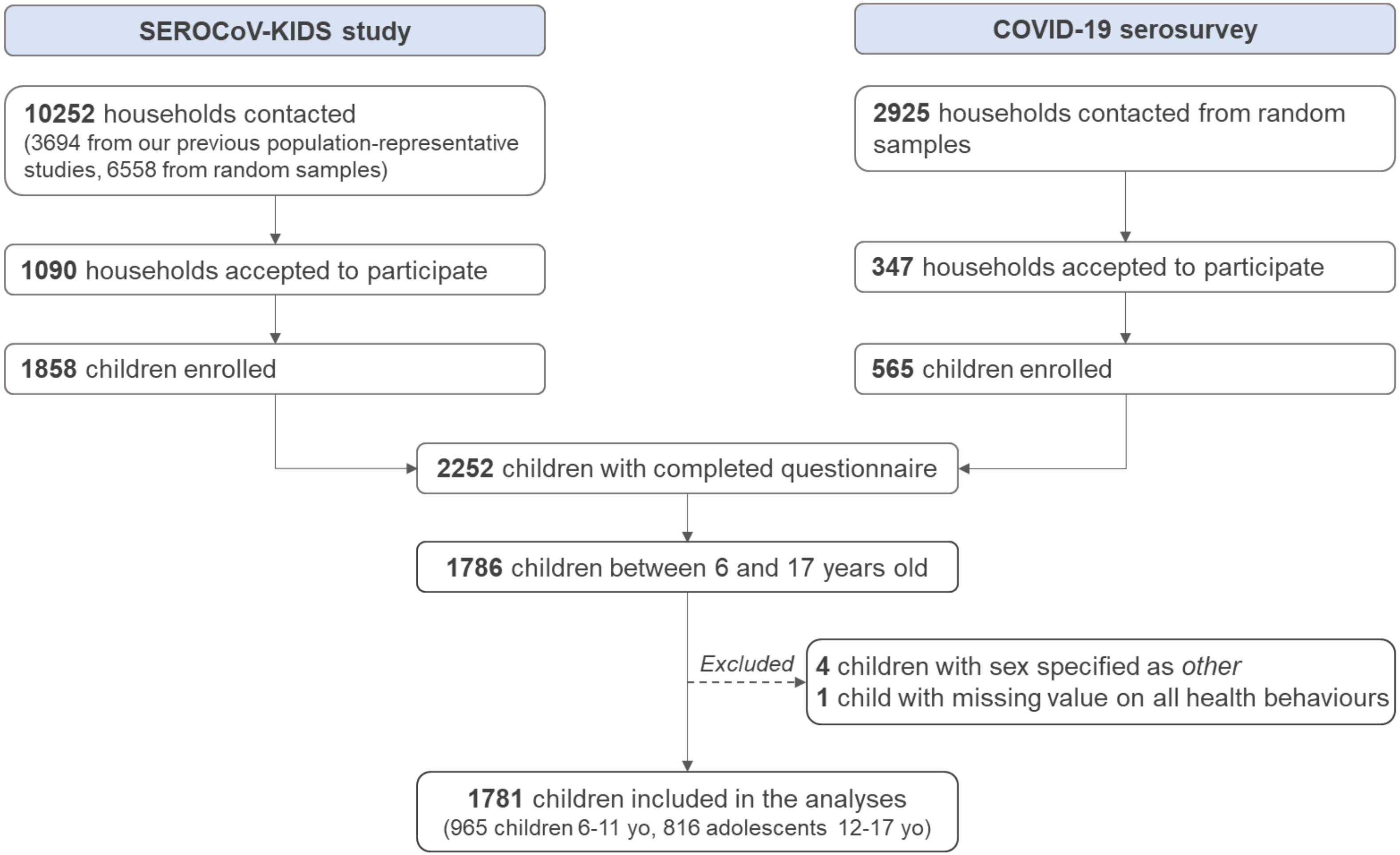

Supplementary figure 1. Selection of study participants

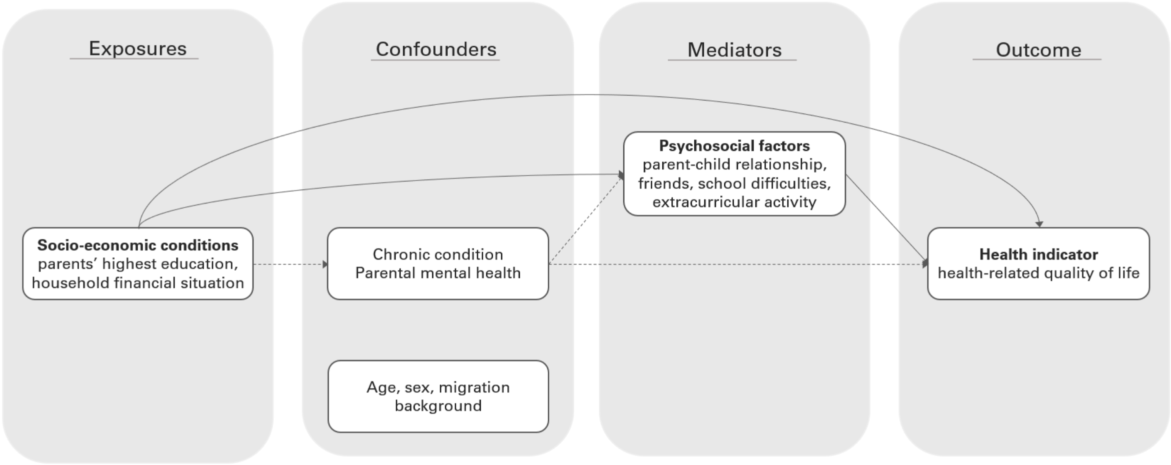

Supplementary figure 2. Directed acyclic graph (DAG) of the association between socio-economic conditions, psychosocial factors, and health indicator. Dashed lines indicate confounding pathways. Age, sex and migration background are considered to be covariates of all associations; arrows are not drawn for readability.

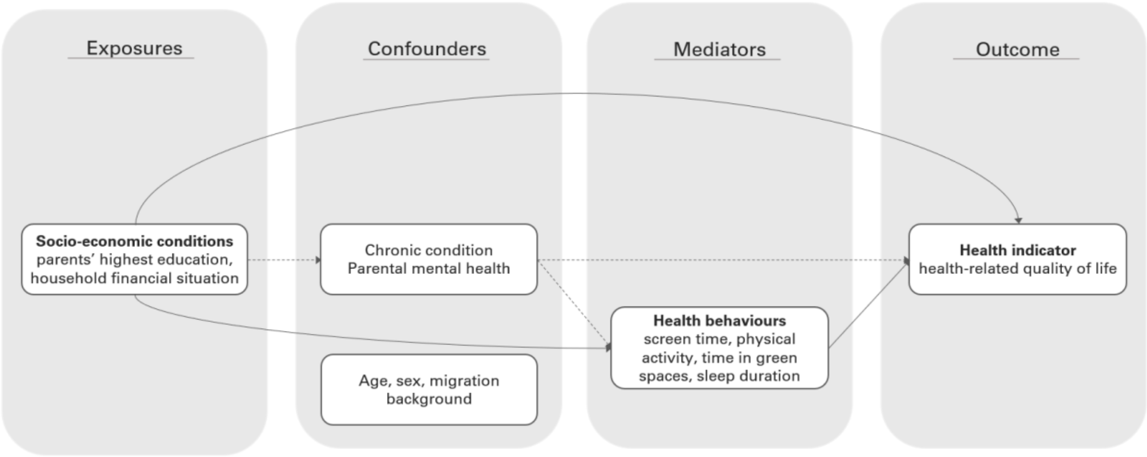

Supplementary figure 3. Directed acyclic graph (DAG) of the association between socio-economic conditions, health behaviours, and health indicator. Dashed lines indicate confounding pathways. Age, sex and migration background are considered to be covariates of all associations; arrows are not drawn for readability.

Supplementary table 1. Summary of the recoding of psychosocial, behavioural and health variables into favourable and unfavourable factors

Age (years)	Favourable	Unfavourable
PSYCHOSOCIAL MEDIATORS		
**Parent-child relationship**		
In general, how would you assess your relationship with the child?		
6-17	good	rather good, average, rather poor, poor
**Number of close friends**		
Does the child have close friends (e.g. friends they frequently spend time with or play with at school or outside of school)?		
6-17	more than three, two to three	one, none
**School difficulties**		
Would you say that the child is experiencing difficulties at school?		
6-17	no, rather no	rather yes, yes
**Extracurricular activity**		
Does the child engage in any extracurricular activity at least once a week?		
6-17	at least one	none
BEHAVIOURAL MEDIATORS		
**Screen time**		
On average, how much time does the child currently spend on screens daily for recreational purposes (e.g. watching videos, gaming or messaging on TV, smartphone, computer)?		
6-13[a]	≤ 2h/day	> 2h/day
14-17[a]	≤ 2h/day (self-report)	> 2h/day (self-report)
**Physical activity**		
On average, how much time does the child currently spend engaging in physical activity daily (e.g., sports, outdoor play, walking, biking)?		
6-17[b]	≥ 1h/day	< 1h/day
**Sleep time**		
On average, how much times does the child sleep daily (total time, including naps)?		
6-13[a]	≥ 9h/day and ≤ 11h/day	< 9h/day or > 11h/day
14-17[a]	≥ 8h/day and ≤ 10h/day	< 8h/day or > 10h/day
**Green spaces time**		
On average, how much times does the child spend in green spaces weekly (e.g. playground, garden, park, forest, mountain)?		
6-17	middle and highest sample tertile	lowest sample tertile
HEALTH OUTCOME		
**Health-related quality of life**		
6-13[c]	PedsQL score ≥ 65.4	PedsQL score < 65.4
14-17[c]	PedsQL score ≥ 69.7 (self-report)	PedsQL score < 69.7 (self-report)

Data was parent-reported unless stated otherwise.

[a] Canadian Society for Exercise Physiology 2016 (see reference nº 32)

[b] World Health Organization 2020 (see reference nº 31)

[c] Varni et al. 2003 (see reference nº 33)

Supplementary table 2. Mediation of psychosocial risk factors and health behaviours in the association between socio-economic conditions and health-related quality of life among children and adolescents

	**Psychosocial risk factors**		**Health behaviours**		**All**	
	**Estimate**	**E-value**	**Estimate**	**E-value**	**Estimate**	**E-value**
	aOR (95% CI)	OR (lower limit of 95% CI)	aOR (95% CI)	OR (lower limit of 95% CI)	aOR (95% CI)	OR (lower limit of 95% CI)
CHILDREN 6-11 years old (n=965)						
**Parents' highest education**						
Direct effect	2.87 (1.65-4.61)	5.18 (2.68)	2.56 (1.45-4.30)	4.55 (2.26)	2.66 (1.47-4.27)	4.76 (2.31)
Indirect effect	1.23 (1.06-1.45)	1.76 (1.31)	1.02 (0.88-1.18)	1.17 (1.00)	1.25 (1.02-1.60)	1.80 (1.14)
Total effect	3.66 (2.13-5.95)	6.77 (3.68)	2.62 (1.53-4.40)	4.68 (2.43)	3.43 (2.00-5.61)	6.32 (3.42)
Proportion mediated (%)	26 (8-45)		3 (-24-26)		28 (2-58)	
**Household financial situation**						
Direct effect	2.32 (1.18-4.76)	4.06 (1.64)	2.93 (1.45-6.28)	5.30 (2.27)	2.25 (1.20-5.31)	3.94 (1.68)
Indirect effect	1.20 (1.04-1.53)	1.69 (1.25)	1.10 (0.96-1.26)	1.43 (1.00)	1.34 (1.08-1.78)	2.01 (1.37)
Total effect	2.84 (1.45-6.34)	5.13 (2.27)	3.23 (1.60-6.74)	5.91 (2.58)	3.13 (1.67-7.76)	5.71 (2.72)
Proportion mediated (%)	25 (5-70)		13 (-6-35)		37 (10-78)	
ADOLESCENTS 12-17 years old (n=816)						
**Parents' highest education**						
Direct effect	1.07 (0.60-1.82)	1.33 (1.00)	1.19 (0.68-1.99)	1.67 (1.00)	0.97 (0.55-1.80)	1.20 (1.00)
Indirect effect	1.37 (1.13-1.69)	2.08 (1.51)	1.08 (0.99-1.20)	1.37 (1.00)	1.49 (1.15-1.89)	2.34 (1.55)
Total effect	1.46 (0.86-2.39)	2.29 (1.00)	1.28 (0.74-2.14)	1.89 (1.00)	1.44 (0.84-2.50)	2.24 (1.00)
Proportion mediated (%)	Undefined[a]		Undefined[a]		Undefined[a]	
**Household financial situation**						
Direct effect	2.36 (1.42-4.58)	4.14 (2.19)	3.53 (1.76-7.38)	6.52 (2.92)	2.69 (1.47-5.50)	4.82 (2.30)
Indirect effect	1.38 (1.14-1.66)	2.11 (1.55)	1.06 (0.96-1.22)	1.33 (1.00)	1.45 (1.12-1.79)	2.27 (1.50)
Total effect	3.34 (1.96-6.24)	6.14 (3.33)	3.76 (1.89-8.37)	6.99 (3.19)	4.05 (2.13-7.86)	7.57 (3.68)
Proportion mediated (%)	40 (18-63)		8 (-8-24)		41 (15-63)	

Results adjusted Odds Ratios (aOR) and 95% confidence intervals from marginal structural models adjusted for age, sex and migration backgound, as well as for chronic condition and parental mental health as mediator-outcome confounders affected by the exposure. Direct, indirect and total effects estimated with logistic marginal structural models from following VanderWeele and Tchetgen Tchetgen, 2017 (see reference nº 38). Proportion mediated calculated as direct effect × (indirect effect – 1) / (total effect – 1). Missing data is handled with multiple imputation and confidence intervals are calculated with bootstraps with 1000 repetitions.

[a] Undefined because of no statistically significant effect of parents' highest education on adolescents' health-related quality of life.
